# Lethal and Sub-Lethal Effects and Modulation of Gene Expression Induced by T Kinase Inhibitors in Zebrafish (Danio Rerio) Embryos

**DOI:** 10.3390/toxics10010004

**Published:** 2021-12-24

**Authors:** Tina Elersek, Matjaž Novak, Mateja Mlinar, Igor Virant, Nika Bahor, Karin Leben, Bojana Žegura, Metka Filipič

**Affiliations:** 1 Department of Genetic Toxicology and Cancer Biology, National Institute of Biology, Večna pot 111, 1000 Ljubljana, Slovenia; tina.elersek@nib.si (T.E.); matjaz.novak@nib.si (M.N.); mateja.mlinar@nib.si (M.M.); zalec.nika@gmail.com (N.B.); leben.karin@gmail.com (K.L.); bojana.zegura@nib.si (B.Ž.); 2 Institute of Oncology Ljubljana, Zaloška 2, 1000 Ljubljana, Slovenia; ivirant@onko-i.si; 3 Biotechnical Faculty, University of Ljubljana, Jamnikarjeva 101, 1000 Ljubljana, Slovenia

**Keywords:** aquatic toxicity, tyrosine kinase inhibitors, zebrafish embryo toxicity test, gene expression, environmental hazard

## Abstract

Tyrosine kinase inhibitors (TKIs) are designed for targeted cancer therapy. The consumption of these drugs during the last 20 years has been constantly rising. In the zebrafish (*Danio rerio*) embryo toxicity test, we assessed the toxicity of six TKIs: imatinib mesylate, erlotinib, nilotinib, dasatinib, sorafenib and regorafenib. Imatinib mesylate and dasatinib induced lethal effects, while regorafenib, sorfenib and dasatinib caused a significant increase of sub-lethal effects, predominantly oedema, no blood circulation and formation of blood aggregates. The analyses of the changes in the expression of selected genes associated with the hormone system after the exposure to imatinib mesylate, dasatinib and regorafenib demonstrated that all three tested TKIs deregulated the expression of oestrogen receptor esr1, cytochrome P450 aromatase (cypa19b) and hydroxysteroid-dehydrogenase (hsd3b), regorafenib, and also thyroglobulin (tg). The expression of genes involved in the DNA damage response (gadd45 and mcm6) and apoptosis (bcl2) was deregulated only by exposure to regorafenib. The data indicate that common mechanisms, namely antiangiogenic activity and interference with steroidogenesis are involved in the TKI induced sub-lethal effects and potential hormone disrupting activity, respectively. The residues of TKIs may represent an environmental hazard; therefore, further ecotoxicological studies focusing also on the effects of their mixtures are warranted.

## 1. Introduction

Aquatic pollution from pharmaceutical residues is a recognized environmental problem [1,2,3]. Of particular concern are residues of cytotoxic anticancer drugs that interact with the DNA function of rapidly dividing cancer cells. Most of these drugs are genotoxic and can cause adverse effects in accidentally exposed aquatic wildlife even at very low concentrations, especially with chronic exposure [4,5,6,7,8].

In the last two decades, cancer treatment has changed from the use of conventional cytotoxic anticancer drugs to therapies targeting specific cancer types with higher specificity and consequently fewer adverse side effects [9,10]. An important group of such drugs are tyrosine kinase inhibitors (TKIs), which are small molecules that target specific kinases that are deregulated in certain cancers. These molecules are designed to inhibit the catalytic activity of the target kinase by blocking the ATP-binding site, thereby preventing phosphorylation of cellular targets involved in cell proliferation [11,12]. However, due to the structural conservation of the ATP-binding site, many TKIs have inhibitory activity against a broader range of protein kinases, with the potential to affect multiple signaling pathways—the so-called off-target activities [13]. Furthermore, signal transduction by tyrosine protein kinases is a general mechanism that is conserved across species, and therefore off-target activities can occur in other exposed organisms. Mammalian cells have a high redundancy of signal transduction pathways that reduce the toxic effects of TKIs towards normal cells. However, in non-mammalian organisms, such redundancy may not be developed and may therefore be more susceptible [14]. 

The first drug in this class is imatinib mesylate (IMAT), which was developed for the treatment of BCR-ABL-associated leukemia and was launched in 2001 [15]. IMAT has been reported as one of the most widely used anticancer drugs [1,16,17]. Currently more than 40 TKIs are approved for cancer treatment [18]. Therefore, with the increasing clinical use of TKIs, the occurrence of residues of these drugs in the aquatic environment is also expected to increase. Moreover, cancer treatment with TKIs is usually long-term or even lifelong and is applied to outpatients, which may further contribute to the increased occurrence of residues of TKIs in the aquatic environment. In Spain (Valencia), IMAT has been detected in hospital wastewater at concentrations of 164 ng/L and in wastewater treatment plant influent at concentrations of 11–577 ng/L [19]. However, no data is available for environmental occurrence of other TKIs. Published ecotoxicological studies showed high toxicity of IMAT to daphnids upon chronic exposure, while algae, higher plants and fish were less sensitive [4,6,20,21,22]. The studies on daphnids [6] and higher plants [20] showed that IMAT has genotoxic potential, which was also confirmed in the in vitro studies with fish and human cell lines [23,24,25]. Moreover, IMAT has estrogenic activity in the E-screen assay system using MDA-MB-231 cells [26]. In a recent study, Novak et al. [22] demonstrated that lifelong chronic exposure of zebrafish to IMAT deregulated the expression of genes associated with steroidogenesis and hormone metabolism and function, suggesting hormone-disrupting activity. Ecotoxicological data are scarce for other TKIs currently in use. For marketing authorization of medicinal products for human use, the European Medicines Agency (EMA) requires an environmental risk assessment based on the use of the product and the physicochemical and ecotoxicological properties of the active substance and its environmental fate [27]. For some TKIs, the environmental risk assessment has been carried out and can be found in European public assessment reports (EPARs) published on the website of the European Medicines Agency (https://www.ema.europa.eu/en/glossary/european-public-assessment-report). These data are generally limited to baseline data on bioaccumulation potential, persistence and acute and subchronic toxicity to aquatic organisms, while there is no information on their potential mechanisms of action.

In this study, we used the zebrafish (*Danio rerio*) embryo toxicity assay (FET test), OECD TG 236 (2013), that allows toxicological evaluation based on multiple endpoints related to lethal and sub-lethal effects. In addition, the zebrafish represents a vertebrate model and the observed effects are relevant not only for predicting adverse effects for fish populations but also for humans. The results of previous comprehensive studies on IMAT, which revealed its potential genotoxic and hormone-disrupting effects, prompted us to comparatively evaluate toxicity and the underlying mechanisms of six TKIs: imatinib mesylate (IMAT), erlotinib (ERLO), nilotinib (NILO), dasatinib (DASA), regorafenib (REGO), and sorafenib (SORA). The TKIs included in the study belong to the group of TKIs most commonly used in cancer treatment and differ in terms of target kinases (Table 1). To gain insight into understanding potential mechanisms of adverse effects of TKIs and their potential hazard to the aquatic environment, IMAT, DASA and REGO were further investigated for their effects on the expression of selected genes involved in hormonal system regulation and DNA damage response pathways in zebrafish embryos. The changes in the gene expression upon exposure to toxic chemicals reflect adaptive processes and/or toxic effect [28] and can be used to predict potential adverse effects of chemicals [29]. 

## 2. Materials and Methods

### 2.1. Consumption of Tyrosine Kinase Inhibitors in Slovenia

Data on consumption of TKIs were obtained from analyzes and archives of prescribed drugs in Slovenia published by the Health Insurance Institute of Slovenia. The data provided contained information on the number of boxes of each drug prescribed in 2010–2019. The amount of the active substance, expressed in [kg], was calculated based on the pack size. From these data, consumption in [kg] per year was calculated for each TKI. The data include only drugs dispensed in public pharmacies, excluding hospital inpatient use, clinical trials, and various compassionate use programs for which data are not available. It is estimated that more than 90% of these drugs are dispensed in public pharmacies.

### 2.2. Test Substances

The selected TKIs were obtained from the following suppliers: IMAT (CAS No. 220127-57-1; MW 589.7 g/mol); ERLO (Cas No. 183321-74-6; MW 393.4 g/mol); and DASA (CAS No. 302962-49-8; MW 488.0 g/mol) from Santa Cruz Biotechnology; NILO (CAS No. 641571-10-0; MW 529.5 g/mol) from Sigma-Aldrich; SORA (CAS No. 284461-73-0; MW 464.8 g/mol) from Toronto Research Chemicals; and REGO (CAS No. 755037-03-7; MW 482.8 g/mol) from Tokyo Chemical Industry Co. As specified by manufacturers, the compounds were of 97% or higher purities. Stock solutions: IMAT 50 mg/L in ddH2O, ERLO 100 g/L in DMSO; NILO 10 g/L in DMSO; SORA 200 g/L in DMSO, DASA 50 g/L in DMSO, and REGO 10 g/L in DMSO were stored at 4 °C for no longer than 1 month. The positive control 3,4-dichloroaniline (3.4-DCA) was supplied from Sigma-Aldrich.

### 2.3. Maintenance of Zebrafish

Wild type zebrafish (TU strain) obtained from (Tubingen, Germany) were maintained under standard laboratory conditions of 26 ± 1 °C on a 12:12 h light: dark cycle in a closed system with a mixture of filtered tap water and distilled water. Fish were fed live arthemia twice daily. Zebrafish embryos used in the experiments were obtained from spawning adult fish kept in groups of approximately 20 males and 20 females. Spawning was induced in the morning when the lights were turned on. Fertilized eggs were collected no later than 1 h after light onset, washed and rinsed several times with ISO water (OECD TG 236). Embryos were examined under inverted microscope (Nikon Eclipse TE300, Tokyo, Japan), and embryos that developed normally and did not exceed the 16-cell stage were selected for subsequent TKI exposure experiments.

### 2.4. TKIs Solutions 

Stock solutions of the TKIs prepared in the solvent DMSO (dimethyl sulfoxide, Riedel-de Haën) were diluted with aerated ISO water (OECD TG 236) to obtain working concentrations. DMSO in the final solution was adjusted to the same concentration, which was 0.5% for ERLO; 0.6% for NILO; 0.03% for SORA; 0.06% for REGO; and 0.5% (*v*/*v*) for DASA. All experiments included the negative control (ISO water) and the solvent control (with maximum % (*v*/*v*) DMSO used for the selected TKIs) and the positive control 3,4- DCA at a final concentration of 4 mg/L in ISO water (OECD TG 236). Prior to the testing, we confirmed that DMSO up to 0.6% (*v*/*v*) did not induce lethal or sub-lethal effects in zebrafish embryos after 24, 48, 72 or 96 h of exposure (data not shown).

The FET assay was performed with at least five concentrations in 1:3.3 serial dilutions. The highest tested concentration of each compound was established based on the solubility in the treatment medium. The following concentrations in mg/L for each TKI were tested: ERLO 1.3, 4.5, 14.7, 48.5, 160.0 mg/L; IMAT 2.0, 6.7, 21.9, 72.4, 239.0 mg/L; NILO 0.6, 1.9, 6.2, 20.3, 67.0 mg/L; DASA 0.5, 1.7, 5.5, 18.2, 60.0 mg/L; REGO 0.05, 0.17, 0.55, 1.82, and 6.0 mg/L; and SORA 0.5, 1.7, 5.5, 18.2, 60.0 mg/L.

### 2.5. Fish Embryo Toxicity Test (FET)

Experiments were conducted as described in the OECD TG 236 (2013) guideline, with the addition of observation of sub-lethal and teratogenic effects. Briefly, all TKIs were tested at 5 to 6 different concentrations in at least three independent experiments, with 20 embryos per concentration in each experiment. Positive control 3,4-DCA (PC), negative control (ISO water) and solvent control (ISO water with maximum DMSO concentration used) were included in each of the independent experiments. Each plate also had an internal negative control (4 wells per plate). The exposure was performed under static conditions. During the experiments, the following conditions were maintained: 12-h light/12-h dark cycle, temperature 26 ± 1 °C, pH 6.8–7.8, oxygen 80–100%. OECD TG 236 aims to determine lethality based on 4 observations (embryo coagulation, somite abnormalities, tail detachment and absence of heartbeat) for the determination of an LC_50_. As sub-lethal endpoints, we evaluated: no spontaneous movement, lack of blood circulation, oedema, blood aggregation, or no pigmentation. As developmental effects including teratogenic effects, we evaluated tail malformation or developmental delay and hatching rates. All observations were made after 24, 48, 72 and 96 h of exposure. According to the OECD protocol, experiments were considered valid if embryo fertilization was greater than 70%, hatching rate after 96 h was greater than 80%, normal development of negative and solvent controls was greater than 90%, and normal development of positive controls was less than 30%.

### 2.6. Gene Expression Analysis

IMAT, DASA and REGO were selected for further transcriptomic analysis. REGO and DASA were tested at concentrations with no observed adverse effect (NOAEC) and low observed adverse effect (LOAEC) determined by the FET assay (taking into account sublethal effects after 96h, including all embryos, not just alive) as follows: REGO (0.2 and 0.6 mg/L), DASA (0.2 and 0.6 mg/L). IMAT was tested at 2.3 and 210 mg/L, representing the lowest concentration tested and the lowest concentration at which a significant increase in lethal effects was observed. For gene expression analysis, 10 embryos per experimental point were exposed to the selected TKIs at two concentrations under the conditions described above (Section 2.4). Experiments were repeated in three independent biological replicates. Pools of five embryos were randomly collected after 96 h of exposure and total RNA was extracted from the whole embryos using TRIzol reagent (Sigma-Aldrich, USA) according to the manufacturer’s protocol. In addition, embryos were mechanically homogenized once in Trizol reagent. Glycogen (20 μg/mL) was added to the cell lysate. The RNA was incubated with isopropyl alcohol overnight at −20 °C to precipitate it. All solutions required for RNA isolation were prepared in RNase-free water. Total RNA content was determined by measuring absorbance at 260 nm, and quality was checked by measuring the 260/280 nm ratio. One percent agarose-formaldehyde gel electrophoresis with ethidium bromide staining was used to further check the quality of total RNA. RNA was transcribed into cDNA using 1 µg of total RNA and the cDNA High Capacity Kit according to the manufacturer’s protocol under the following conditions: 4 cycles of 40 min (1 step at 25 °C and 3 steps at 37 °C).

The mRNA expression of the selected genes was quantified using 48 Dynamic Array™ IFC chips for gene expression on the BioMark HD instrument system (Fluidigm, UK). PCR conditions were 50 °C for 2 min, 95 °C for 10 min and 40 cycles of 95 °C for 15 s and 60 °C for 1 min. The following selected genes were pre-amplified: tg (thyroglobulin), Dr03986645_m1; tshba (thyroid stimulating hormone subunit beta a), Dr03150633_m1; thrb (thyroid hormone receptor beta); Dr03138254_m1; thraa (thyroid hormone receptor alpha a), Dr03131485_m1; dio2 (deiodinase, iodothyronine, type II), Dr03088603_m1; cyp51 (cytochrome P450, family 51), Dr03114750_m1; cyp19a1b (cytochrome P450, family 19, subfamily A, polypeptide 1b), Dr03080043_m1; hsd3b2 (hydroxy-delta-5-steroid dehydrogenase, 3 beta- and steroid delta-isomerase 2), Dr03133230_m1; hsd17b3 (hydroxysteroid (17-beta) dehydrogenase 3), Dr03074978_m1; esr2b (estrogen receptor 2b), Dr03150586_m1; esr1 (estrogen receptor 1) Dr03093579_m1; apoeb (apolipoprotein Eb); Dr03124984_m1; gadd45ab (growth arrest and DNA damage inducible, alpha, b), Dr03425943_m1; mcm6 (minichromosome maintenance complex 6), Dr03130479_m1; dnajb9a (DnaJ heat shock protein family (Hsp40) member B9a); Dr03122595_m1; bcl2a (BCL2 apoptosis regulator a), Dr03091049_m1; pax2a (paired box gene 2a), Dr03138228_m1; slc35b1 (solute carrier family 35 member B1); Dr03102246_m1; and Nkx2.4b (NK2 homebox 4b), Dr03118927_m1. In all experiments, B-actin (Dr03432610_m1) was used as a reference gene. Contamination with genomic DNA was controlled by no-RT controls. For relative quantification of gene expression, the standard curve method was used and transcript accumulation of each gene was normalized to B-actin gene expression. Open-source QuantGenious web software was used to analyze the data (relative quantification according to the appropriate solvent control) [30].

### 2.7. Statistical Evaluations 

Results expressed as % of effects were analysed using Prism 6 software (GraphPad Inc., California, CA, USA). For lethal, sub-lethal and teratogenic effects, data were analysed as measurements from each well (pooled together) rather than as means of replicates to extract as much information as possible (OECD, 2013). For the plots showing % of effect vs. concentration of TKIs, a "log(agonist) vs. response-find EC anything" nonlinear regression model was applied (Equations (1)−(3)). The residual plots were also examined, and any outlier detected by Prism was excluded from the statistical analyses. Statistical significance (*p* 0.05) of an effect compared to the solvent control was assessed using a nonparametric ANOVA (Kruskal–Wallis test) with Dunnett’s post-test at a 95% confidence interval.

(1) $$logECF\;=\;logEC50\;+\;\left(\frac{1}{slope}\right)\;\times \;log\left(\frac{F\%}{100\;-\;F\%}\right)\;$$

(2) $$F\%\;=\;\frac{Y\;-\;bottom}{\left(top\;-\;bottom\right)\;\times \;100}\;$$

(3) $$Y\;=\;bottom\;+\;\left(\frac{top\;-\;bottom}{1\;+\;10\left(\left(log50\;-\;X\right)\;\times \;slope\right)}\right)$$

Statistical analysis of gene expression results was performed using a two-tailed Student’s *t*-test. The expression of target genes in treated embryos was divided by the average expression of the corresponding vehicle control samples for each treatment separately. Only differences greater than 1.5-fold were considered up/down-regulation (relative expression > 1.5 or <0.66, respectively).

## 3. Results and Discussion

Data on the potential adverse effects of unintentional environmental exposure of aquatic biota to TKI residues are very sparse, which prevents adequate hazard assessment. In this study, we evaluated the toxicity of six of the most commonly used TKIs using the zebrafish embryo toxicity assay. The results showed a differential response of zebrafish embryos to the six TKIs in terms of lethal and sub-lethal concentrations, while the types of sub-lethal changes observed were similar and related to their mechanism of therapeutic action. The changes in gene expression induced by the TKIs tested indicated a hormone disrupting potential of the tested TKIs.

### 3.1. Consumption of TKIs in Slovenia

**Figure 1:**
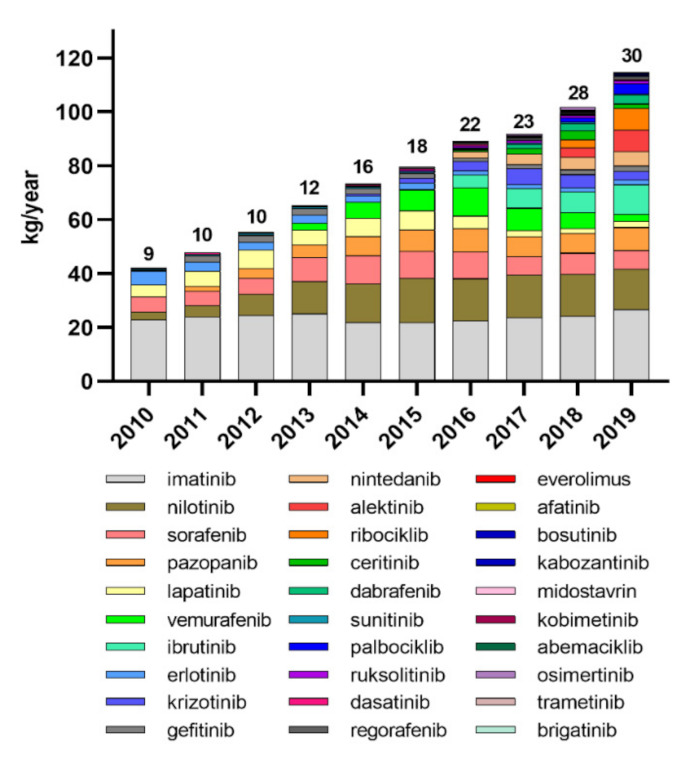
Consumption of tyrosine kinase inhibitors in Slovenia from 2010 to 2019. The numbers above the columns represent the number of registered TKIs per year.

The production and use of TKIs for the targeted cancer therapy is constantly increasing worldwide [31]. We analyzed the data from national reports on the consumption of TKIs dispensed from public pharmacies in Slovenia in the last 10 years. The data clearly show that the number of registered TKIs has increased from nine in 2010 to thirty in 2019, and the total consumption of these drugs has almost tripled: from 42 kg/year to 116 kg/year (Figure 1). Consumption of older TKIs, such as IMAT and ERLO, has stagnated or even decreased, while consumption of most new approved TKIs is increasing. Although the data only represent consumption in Slovenia, similar trends of increasing consumption of TKIs can be expected in other developed countries. Consequently, we can expect an increase in the occurrence of residues of various TKIs in the environment, and consequently, organisms in the environment can be expected to be exposed to increased concentrations of complex mixtures of the residues of these drugs.

### 3.2. Fish Embryo Toxicity Test 

**Figure 2:**
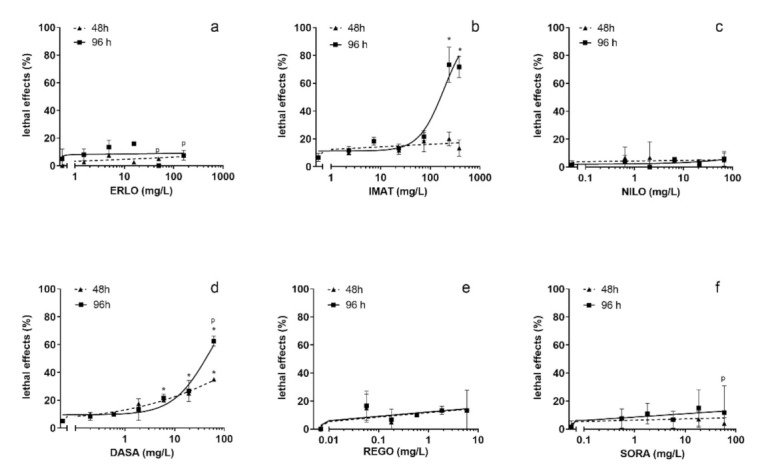
Lethality of the selected tyrosine kinase inhibitors in zebrafish embryo test after 48 and 96 h exposure. ERLO was tested in the concentration range 1.9–200 mg/L (**a**), IMAT 2.3–239 mg/L (**b**), NILO 0.6–67 mg/L (**c**), DASA 0.6–60 mg/L (**d**), REGO 0.06–6 mg/L (**e**), and SORA 0.6–60 mg/L (**f**). * indicates the statistical difference compared to the negative control (tested with ANOVA). p indicates the precipitation of the compound in the treatment medium.

Exposure to ERLO, NILO, REGO and SORA did not cause a statistically significant increase in lethal effects (Figure 2a,c,e,f). Exposure to IMAT for 96 h induced a dose-dependent increase in the number of coagulated embryos with a steep increase at concentrations ≥ 210 mg/L (Figure 2b). DASA induced a dose-dependent increase in the number of coagulated embryos at 48 and 96 h, which was significant at concentrations ≥ 0.6 mg/L (Figure 2d). The LC_50_ values for IMAT and DASA after 96 h exposure were 251 (205–307) mg/L and 51 (39–67) mg/L, respectively. It should be noted that the TKIs were tested at different concentration ranges selected on the basis of their solubility in the treatment medium. Therefore, LC_50_ values could only be determined for IMAT and DASA.

In our previous study of IMAT with the zebrafish embryo toxicity test, the LC_50_ value at 96 h exposure was lower (118 mg/L) [32], but still in the same range as the LC_50_ obtained in this study. In contrast to our study, where REGO and SORA did not cause lethal effects, Chimote et al. reported LC_50_ ≤ 1 mg/L after 96 h for both [33]. In another study, the LC_50_ for SORA was 2.5 mg/L after 120 h exposure [34]. However, it should be noted that these studies aimed to investigate the antiangiogenic efficacy of TKIs and compare the therapeutic indexes, respectively, and therefore the experimental conditions differed from ours. For example, in both studies, the exposure medium was replaced with fresh every day, whereas our study was conducted under static exposure conditions. The tested drugs have high bioaccumulation potential. The log K_ow_ values are in a range from 3.5 to 4.5, while bioaccumulation in fish is for ERLO 7.8–10 BCF L/kg, for SORA 7250 BCF L/kg, and REGO 2018-3241 BCF L/kg (data from EPAR: https://www.ema.europa.eu/en/glossary/european-public-assessment-report). The much higher toxicity of REGO and SORA in the experiments in which the medium containing the tested compounds was replaced daily can be explained by the high bioaccumulation potential of these two TKIs. 

**Figure 3:**
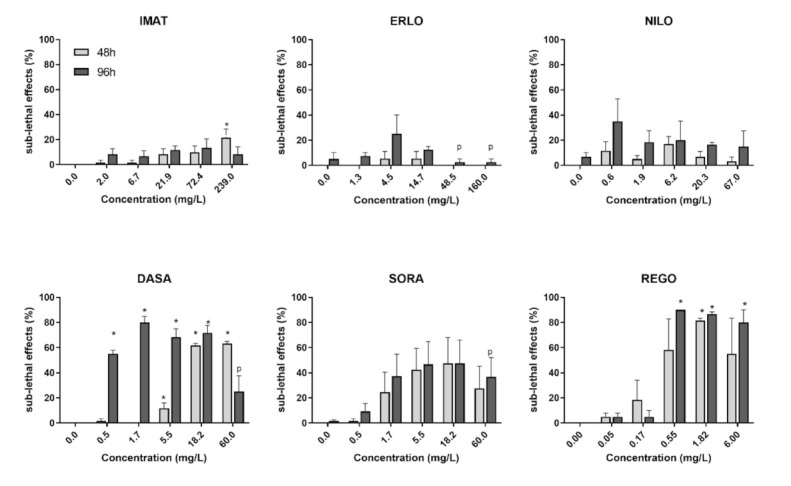
Sub-lethal effects of the selected tyrosine kinase inhibitors after 48 (light grey) and 96 h exposure (dark grey). Results are presented as mean percentage (±SE) of sub-lethal and teratogenic effects from three independent experiments. Percentage of sub-lethal effect and statistical comparisons were calculated considering all embryos per drug concentration (60 embryos from three experiments). A statistically significant increase in the percentage of sub-lethal effects compared to the negative control (ANOVA) is indicated by *. The letter p indicates precipitation of the compound in the treatment medium.

**Figure 4:**
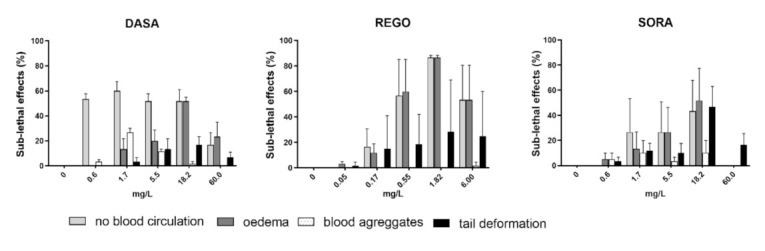
The incidence (%) of observed sub-lethal (lack of blood circulation, oedema and blood aggregates) and teratogenic effects (tail deformity) in zebrafish embryos after 96 h of exposure to SORA, REGO and DASA. Percentage of sub-lethal effect and statistical comparisons were calculated considering all embryos per drug concentration (60 embryos from three experiments). Data are presented as mean percentage (±SE) of observed sub-lethal effects from three independent experiments.

The highest frequency of sub-lethal effects was observed in embryos exposed to REGO followed by DASA and SORA, while ERLO, NILO and IMAT did not induce a significant increase in sub-lethal or teratogenic effects compared to the control (Figure 3). After 96 h, DASA, REGO, and SORA predominantly induced oedema and lack of blood circulation, whereas DASA and SORA also induced blood aggregate formation (Figure 4). In embryos exposed to REGO, DASA, SORA and IMAT for 96 h, tail deformation and growth retardation were also observed (Figure 4), indicating a teratogenic effect. After 96-h exposure, the majority of embryos exposed to REGO, SORA and DASA suffered multiple sub-lethal effects associated with low embryo survival [6].

The observed formation of oedema, lack of blood circulation, and formation of blood aggregates in the zebrafish embryo may be related to the antiangiogenic activity of TKIs. Several previous studies [33,35] have reported similar sub-lethal effects of REGO and SORA, both of which act on the vascular endothelial growth factor (VEGF) signaling pathway involved in angiogenesis. In our study, a similar pattern of sub-lethal effects as REGO and SORA was also induced by DASA, which affects Src family tyrosine kinases that regulate several events involved in angiogenesis [36]. Clinical studies have shown that treatment of patients with TKIs is associated with cardiotoxicity, including cardiomyopathy, hypertension, arterial and venous thrombosis, which are the consequence of the influence of TKIs on normal vascular physiology [37]. The observed induction of oedema, lack of blood circulation, and blood aggregates by DASA, REGO, and SORA in zebrafish embryos, may thus reflect the side effects observed in patients.

**Figure 5:**
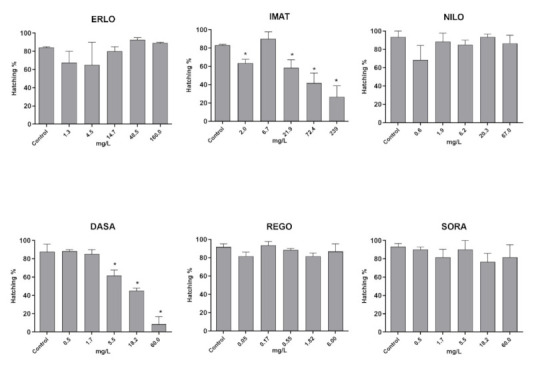
Hatching rate in zebrafish embryos after 96 h exposure to the selected TKI. Results are presented as mean values of percentage of embryos hatched ± SD. Percentage of hatched embryos and statistical comparisons were calculated considering all embryos per drug concentration (60 embryos from three experiments). Statistical difference from the control (ANOVA) is indicated by *.

The reduction in hatching rate is considered a sub-lethal effect with significant implications for population viability in the wild. The hatching rate was significantly reduced after exposure to IMAT and DASA, while the other four TKIs tested did not affect it (Figure 5).

Taken together, DASA, REGO and SORA that target multiple kinases induced more severe sub-lethal effects than ERLO, IMAT and NILO targeting only few kinases (Table 1).

### 3.3. Modulation of Gene Expression

There is evidence from clinical trials that side effects associated with the use of TKIs include thyroid and adrenal dysfunction, bone remodeling, and gonadal dysfunction [38,39,40]. However, experimental data on their hormone disrupting activity in the context of non-intentional environmental exposure are rather limited. IMAT has been shown to exhibit endocrine disrupting activity (estrogenic activity) in vitro [26]. In a recent study of toxicity from chronic exposure across the generation using zebrafish, we demonstrated that IMAT deregulates the expression of a number of genes associated with steroidogenesis and hormone metabolism and function, as well as certain genes involved in the DNA damage response in adult fish [22]. In addition, IMAT has been shown to cause DNA damage in non-target environmental organisms in plants [20,41] and daphnids [6], and mammalian and fish cells in vitro [23,24], but not in vivo in adult zebrafish [22]. To gain insight into the potential endocrine disrupting activity and genotoxicity of TKIs, we investigated the influence of REGO, DASA and IMAT on the expression of selected genes related to the hypothalamic-pituitary-thyroid (HPT) axis, steroidogenesis, oestrogen signaling and DNA damage response pathways during early embryonic development. The three TKIs demonstrated differences in the induced effect in embryos. IMAT induced lethal but not sub-lethal effects, DASA induced lethal and sub-lethal effect, and REGO induced only sub-lethal effects.

The results showed that all three TKIs tested deregulated the expression of *esr1*, *cyp19a1b* and *hsd3b* (Table 2). The oestrogen receptor (esr1) plays a fundamental role in ovarian maintenance [42] and its deregulation may have consequences for the reproduction. In the zebrafish whole-generation study, IMAT downregulated *esr2* in females [22]. Cytochrome P450 aromatase (cyp19) is responsible for the conversion of androgens to estrogens, which play a critical role in developmental sex differentiation in vertebrates [43]. Deregulation of *cyp19* gene expression in zebrafish has been reported following exposure to numerous synthetic and natural endocrine disruptors and has been suggested to be an excellent transcriptional marker for endocrine disruption [44]. Hsd3 catalyses the synthesis of δ4-steroids in the adrenal glands and gonads and has been shown to have a morphogenetic function during the early phase of zebrafish development [45]. Deregulation of these three genes could lead to alterations in androgens and thus disruption of sexual differentiation and early development. In embryos exposed to DASA, the expression of hsd3b was upregulated at the concentration that did not induce adverse effects, while at the concentration that induced sub-lethal effects its expression was attenuated. Such observations are not uncommon as at non-toxic and toxic concentrations the gene expression regulation pathways may be differently affected. REGO and IMAT upregulated the expression of *cyp51*, which encodes sterol 14a-demethylase that plays an essential role in sterol biosynthesis [46]. *Cyp51* was also upregulated by exposure to IMAT in the zebrafish whole-generation toxicity study [22]. REGO also upregulated the gene encoding thyroglobulin (*tg*), which is a precursor to the thyroid hormones T4 and T3. Changes in *tg* expression are commonly used as a marker to monitor thyroid activity during development [47,48]. In fish development, thyroid hormones (THs) play a role in mediating the metamorphic transition from larval to adult stages and influence the maturation of tissues such as bones, gonads, gut and the central nervous system [49,50]. The changes in TH levels, especially during sensitive developmental windows, can have significant acute and potentially long-term adverse effects. Taken together, the gene expression analyses demonstrated the potential of the tested TKIs to disturb regulation of hormone function in zebrafish embryos that can have long-term consequences during their development. These data provide important new information that is highly relevant also in the context to the EMA Guideline on the environmental risk assessment of medicinal products for human use (2006) that requires environmental risk assessment irrespective of the quantity released into the environment.

Changes in the expression of the DNA damage-responsive genes *gadd45a, mcm6*, and *bcl2* were observed only in embryos exposed to REGO (Table 2). Gadd45a plays a role in cell cycle control in the G2-M checkpoint, DNA repair process and apoptosis [51,52]. It is induced by DNA damage and growth arrest signals [53]. The MCM proteins are helicases that interact with S-phase checkpoint regulators, and with components of DNA repair pathways [54]. The upregulation of *gadd45a* and *mmc6* indicate that REGO induced growth arrest signals that may be activated in response to DNA damage. The *bcl2* gene belongs to the family of anti-apoptotic genes that maintain cell survival by inhibiting pro-apoptotic genes [55]. The expression of *bcl2* was downregulated indicating the activation of apoptotic processes. Indeed, activation of apoptosis in cancer cells is one of the mechanisms of anti-cancer effect of TKIs. The studies have shown that REGO induces apoptosis in colon and bladder cancer via suppressing the activation of nuclear factor kappa B (NF-κB) [56,57]. Although induction of apoptosis in cancer cells is a desirable effect in normal tissues and especially during embryonic development, deregulation of apoptotic processes can lead to various developmental defects [58].

## 4. Conclusions

The study showed low toxicity of the tested TKIs in the zebrafish embryo test. IMAT and DASA induced lethal effects, while REGO, SORA and DASA caused a significant increase of sub-lethal effects, predominantly oedema, no blood circulation and formation of blood aggregates. Transcriptomic analysis revealed that IMAT, REGO and SORA deregulated the steroidogenic pathway and oestrogen signaling, which play a role in early-stage development of the zebrafish and may potentially lead to delayed adverse effects. These data indicate that common mechanisms are involved in the sub-lethal effects and potential hormone disrupting activity induced by the TKIs, namely antiangiogenic activity and interference with steroidogenesis, respectively, which suggests possible additive effects of mixtures of the residues of these drugs. Only REGO deregulated expression of genes associated with DNA damage response indicating potential genotoxic activity. Although the effects were observed at high concentrations that are not expected in the environment, the results obtained gave important insight into the mechanisms of adverse effects and indicate that TKIs should be considered hazardous to aquatic organisms. However, for a reliable environmental risk assessment, we would need further ecotoxicological data focusing also on the effects of mixtures of these compounds as well as information on the occurrence of TKI residues in the aquatic environment, which is currently almost completely lacking. On the other hand, zebrafish have many similar characteristics to mammals including humans, and are increasingly used as experimental models in drug development and toxicology to predict effects in humans. In this context, the results of this study, especially the gene expression analyses, may also be relevant for predicting or explaining the adverse effects of TKIs in treated patients.

## Figures and Tables

**Table 1:** Selected tyrosine kinase inhibitors and their kinase targets.

Compound	Kinase Target
Imatinib mesylate	BCR-ABL, PDGFR, c-KIT
Erlotinib	EGFR
Nilotinib	BCR-ABL, PDGFR, DDR1
Sorafenib	VEGFR2, PDGFR, KIT, FLT3, BRAF
Dasatinib	BCR-ABL, SRC family (SRC, LCK, YES, FYN), c-KIT, EPHA2, PDGFRβ
Regorafenib	VEGFR, PDGFR, BRAF, RAF-1, KIT, Ret, TIE-2, FGFR

Note: BCR-ABL, breakpoint cluster region gene-Abelson proto-oncogene fusion protein; EGFR, epidermal growth factor receptor; SRC, proto-oncogene tyrosine-protein kinase Src; LCK, lymphocyte cell-specific protein-tyrosine kinase; YES, proto-oncogene tyrosine-protein kinase Yes; FYN, proto-oncogene tyrosine-protein kinase Fyn; c-KIT, proto-oncogene tyrosine-protein kinase Kit; EPHA2, ephrin type-A receptor 2; PDGFR (α/β), platelet-derived growth factor receptor (α/β); BTK, Bruton’s tyrosine kinase; DDR1, discoidin domain receptor family, member 1; VEGFR (1/2/3), vascular endothelial growth factor receptor (1/2/3); RAF, proto-oncogene serine/threonine-protein kinase; RET, proto-oncogene tyrosine-protein kinase receptor Ret; FGFR-1/2, fibroblast growth factor receptor 1/2; TIE-2, angiopoietin-1 receptor; FLT3, fms-like tyrosine kinase 3; BTAF, serine-threonine kinase.

**Table 2:** Effects of IMAT, REGO and DASA on mRNA expression of selected genes in exposed zebrafish embryos. Results are expressed as relative mRNA expression levels normalized to the corresponding control. Data are means ± SD of three independent experiments with two technical duplicates. Significant differences (*p* ≤ 0.05) between treated and non-treated embryos are marked with *. Genes that were deregulated ≥ 1.5-fold compared to the corresponding control are marked in bold.

Gene	REGO		DASA		IMAT	
Symbol	0.2 mg/L	0.6 mg/L	0.2 mg/L	0.6 mg/L	2.3 mg/L	210 mg/L
***tg***	1.67 ± 0.18*	2.09 ± 0.67 *	1.26 ± 0.37	0.74 ± 0.18	0.72 ± 0.01 *	1.27 ± 0.33
*tshb*	0.99 ± 0.12	0.92 ± 0.13	1.02 ± 0.13	0.98 ± 0.18	0.95 ± 0.07	1.16 ± 0.19
*thrb*	0.99 ± 0.12	0.85 ± 0.14	0.91 ± 0.12	0.88 ± 0.08	0.94 ± 0.09	0.98 ± 0.16
*thraa*	1.05 ± 0.08	0.98 ± 0.12	1.00 ± 0.07	0.91 ± 0.12	0.86 ± 0.14	0.92 ± 0.08
*dio2*	0.95 ± 0.13	0.69 ± 0.15 *	1.05 ± 0.23	1.11 ± 0.06	0.94 ± 0.19	0.74 ± 0.18
***cyp51***	1.14 ± 0.36	2.35 ± 0.43 *	1.07 ± 0.39	1.13 ± 0.18	1.17 ± 0.05	1.75 ± 0.30 *
***cyp19a1b***	4.43 ± 1.06 *	4.35 ± 1.17 *	0.96 ± 0.30	**1.65 ± 0.67**	**1.78 ± 0.73**	2.36 ± 0.55 *
***hsd3b***	2.21 ± 0.67 *	2.04 ± 1.04 *	1.89 ± 0.39 *	0.66 ± 0.03 *	0.58 ± 0.13 *	1.21 ± 0.35
*hsd17b3*	1.06 ± 0.39	1.15 ± 0.21	1.07 ± 0.43	0.89± 0.08	1.31 ± 0.65	1.32 ± 0.20
*esr2b*	1.09 ± 0.03	0.89 ± 0.15	1.07 ± 0.43	0.98 ± 0.08	1.07 ± 0.29	1.16 ± 0.30
*esr1*	0.52 ± 0.02 *	0.38 ± 0.07 *	0.68 ± 0.10	0.63 ± 0.16 *	**0.66 ± 0.15**	**0.65 ± 0.20**
*apoeb*	0.86 ± 0.34	1.13 ± 0.52	1.13 ± 0.41	0.82 ± 0.16	0.94 ± 0.06	0.96 ± 0.30
***gadd45***	1.53 ± 0.31 *	0.95 ± 0.37	1.14 ± 0.42	1.10 ± 0.52	0.86 ± 0.05	1.01 ± 0.10
***mcm6***	0.86 ± 0.14	0.64 ± 0.11 *	0.96 ± 0.11	0.89 ± 0.07	0.78 ± 0.17	0.78 ± 0.05
*dnajb9*	0.89 ± 0.11	0.74 ± 0.02	0.97 ± 0.03	0.84 ± 0.06	1.05 ± 0.15	1.03 ± 0.10
***bcl2***	0.95 ± 0.18	1.52 ± 0.20 *	1.02 ± 0.03	0.72 ± 0.05 *	0.95 ± 0.14	0.90 ± 0.17
*pax2a*	1.19 ± 0.39	1.09 ± 0.37	1.03 ± 0.45	0.73 ± 0.001	0.95 ± 0.29	1.13 ± 0.20
*slc35*	0.87 ± 0.20	0.69 ± 0.13	1.05 ± 0.05	0.86 ± 0.08	0.77 ± 0.18	0.67 ± 0.10
*nkx1*	1.01 ± 0.07	0.73 ± 0.05	1.00 ± 0.11	0.96 ± 0.15	0.80 ± 0.17	0.95 ± 0.32

## Data Availability

The original contribution for this study is included in the article.

## References

[1] Besse J.P., Latour J.F., Garric J. (2012). Anticancer drugs in surface waters: What can we say about the occurrence and environmental significance of cytotoxic, cytostatic and endocrine therapy drugs?. Environ. Int..

[2] Kümmerer K., Haiß A., Schuster A., Hein A., Ebert I. (2016). Antineoplastic compounds in the environment—substances of special concern. Environ. Sci. Pollut. Res..

[3] Kosjek T., Heath E. (2011). Occurrence, fate and determination of cytostatic pharmaceuticals in the environment. TrAC Trends Anal. Chem..

[4] Brezovšek P., Eleršek T., Filipič M. (2014). Toxicities of four anti-neoplastic drugs and their binary mixtures tested on the green alga Pseudokirchneriella subcapitata and the cyanobacterium Synechococcus leopoliensis. Water Res..

[5] Parrella A., Lavorgna M., Criscuolo E., Russo C., Fiumano V., Isidori M. (2014). Acute and chronic toxicity of six anticancer drugs on rotifers and crustaceans. Chemosphere.

[6] Parrella A., Lavorgna M., Criscuolo E., Russo C., Isidori M. (2015). Eco-genotoxicity of six anticancer drugs using comet assay in daphnids. J. Hazard. Mater..

[7] Česen M., Eleršek T., Novak M., Žegura B., Kosjek T., Filipič M., Heath E. (2016). Ecotoxicity and genotoxicity of cyclophosphamide, ifosfamide, their metabolites/transformation products and their mixtures. Environ. Pollut..

[8] Isidori M., Lavorgna M., Russo C., Kundi M., Žegura B., Novak M.M., Filipič M., Mišík M., Knasmueller S., de Alda M.L. (2016). Chemical and toxicological characterisation of anticancer drugs in hospital and municipal wastewaters from Slovenia and Spain. Environ. Pollut..

[9] Besse J.-P., Kausch-Barreto C., Garric J. (2008). Exposure Assessment of Pharmaceuticals and Their Metabolites in the Aquatic Environment: Application to the French Situation and Preliminary Prioritization. Hum. Ecol. Risk Assess. Int. J..

[10] Levitzki A. (2012). Tyrosine Kinase Inhibitors: Views of Selectivity, Sensitivity, and Clinical Performance. Annu. Rev. Pharmacol. Toxicol..

[11] Yamaoka T., Kusumoto S., Ando K., Ohba M., Ohmori T. (2018). Receptor Tyrosine Kinase-Targeted Cancer Therapy. Int. J. Mol. Sci..

[12] Crisci S., Amitrano F., Saggese M., Muto T., Sarno S., Mele S., Vitale P., Ronga G., Berretta M., Di Francia R. (2019). Overview of Current Targeted Anti-Cancer Drugs for Therapy in Onco-Hematology. Medicina.

[13] Liu S., Kurzrock R. (2014). Toxicity of targeted therapy: Implications for response and impact of genetic polymorphisms. Cancer Treat. Rev..

[14] Seiler J.P. (2002). Pharmacodynamic activity of drugs and ecotoxicology—Can the two be connected?. Toxicol. Lett..

[15] Cohen M.H., Williams G., Johnson J.R., Duan J., Gobburu J., Rahman A., Benson K., Leighton J., Kim S.K., Wood R. (2002). Approval Summary for Imatinib Mesylate Capsules in the Treatment of Chronic Myelogenous Leukemia Approval Summary for Imatinib Mesylate Capsules in the Treatment of Chronic Myelogenous Leukemia. Clin. Cancer Res..

[16] Booker V., Halsall C., Llewellyn N., Johnson A., Williams R. (2014). Prioritising anticancer drugs for environmental monitoring and risk assessment purposes. Sci. Total Environ..

[17] Cristóvão M.B., Janssens R., Yadav A., Pandey S., Luis P., Van der Bruggen B., Dubey K.K., Mandal M.K., Crespo J.G., Pereira V.J. (2020). Predicted concentrations of anticancer drugs in the aquatic environment: What should we monitor and where should we treat?. J. Hazard. Mater..

[18] Pottier C., Fresnais M., Gilon M., Jérusalem G., Longuespée R., Sounni N.E. (2020). Tyrosine kinase inhibitors in cancer: Breakthrough and challenges of targeted therapy. Cancers.

[19] Olalla A., Negreira N., de Alda M.L., Barceló D., Valcárcel Y. (2018). A case study to identify priority cytostatic contaminants in hospital effluents. Chemosphere.

[20] Pichler C., Filipič M., Kundi M., Rainer B., Knasmueller S., Mišík M. (2014). Assessment of genotoxicity and acute toxic effect of the imatinib mesylate in plant bioassays. Chemosphere.

[21] Parrella A., Kundi M., Lavorgna M., Criscuolo E., Russo C., Isidori M. (2014). Toxicity of exposure to binary mixtures of four anti-neoplastic drugs in Daphnia magna and Ceriodaphnia dubia. Aquat. Toxicol..

[22] Novak M., Baebler Š., Žegura B., Rotter A., Gajski G., Gerić M., Garaj-Vrhovac V., Bakos K., Csenki Z., Kovács R. (2021). Deregulation of whole-transcriptome gene expression in zebrafish (Danio rerio) after chronic exposure to low doses of imatinib mesylate in a complete life cycle study. Chemosphere.

[23] Novak M.M., Žegura B., Nunić J., Gajski G., Gerić M., Garaj-Vrhovac V. (2017). Assessment of the genotoxicity of the tyrosine kinase inhibitor imatinib mesylate in cultured fish and human cells. Mutat. Res. Genet. Toxicol. Environ. Mutagen..

[24] Novak M., Žegura B., Baebler Š., Štern A., Rotter A., Stare K., Filipič M., Novak M., Baebler Š., Štern A. (2016). Influence of selected anti-cancer drugs on the induction of DNA double-strand breaks and changes in gene expression in human hepatoma HepG2 cells. Environ. Sci. Pollut. Res..

[25] Gajski G., Gerić M., Domijan A.M., Golubović I., Garaj-Vrhovac V. (2019). Evaluation of oxidative stress responses in human circulating blood cells after imatinib mesylate treatment—Implications to its mechanism of action. Saudi Pharm. J..

[26] Parrella A., Lavorgna M., Criscuolo E., Russo C., Isidori M., Russo C., Parrella A., Criscuolo E. (2014). Estrogenic activity and cytotoxicity of six anticancer drugs detected in water systems. Sci. Total Environ..

[27] Whomsley R., Brendler-Schwaab S., Griffin E., Jensen J., Moermond C., Scholz B., Nilssen L.S., Stemplewski H., Roennefahrt I. (2019). Commentary on the draft revised guideline on the environmental risk assessment of medicinal products for human use. Environ. Sci. Eur..

[28] Girardot F., Monnier V., Tricoire H. (2004). Genome wide analysis of common and specific stress responses in adult drosophila melanogaster. BMC Genom..

[29] Williams T.D., Mirbahai L., Chipman J.K. (2014). The toxicological application of transcriptomics and epigenomics in zebrafish and other teleosts. Brief. Funct. Genom..

[30] Baebler Š., Svalina M., Petek M., Stare K., Rotter A., Pompe-Novak M., Gruden K. (2017). quantGenius: Implementation of a decision support system for qPCR-based gene quantification. BMC Bioinform..

[31] Hill A., Gotham D., Fortunak J., Meldrum J., Erbacher I., Martin M., Shoman H., Levi J., Powderly W.G., Bower M. (2016). Target prices for mass production of tyrosine kinase inhibitors for global cancer treatment. BMJ Open.

[32] Kovács R., Bakos K., Urbányi B., Kövesi J., Gazsi G., Csepeli A., Appl Á.J., Bencsik D., Csenki Z., Horváth Á. (2016). Acute and sub-chronic toxicity of four cytostatic drugs in zebrafish. Environ. Sci. Pollut. Res..

[33] Chimote G., Sreenivasan J., Pawar N., Subramanian J., Sivaramakrishnan H., Sharma S. (2014). Comparison of effects of anti-angiogenic agents in the zebrafish efficacy-toxicity model for translational anti-angiogenic drug discovery. Drug Des. Dev. Ther..

[34] Lin H.-S., Huang Y.-L., Wang Y.-R.S., Hsiao E., Hsu T.-A., Shiao H.-Y., Jiaang W.-T., Sampurna B.P., Lin K.-H., Wu M.-S. (2019). Identification of Novel Anti-Liver Cancer Small Molecules with Better Therapeutic Index Than Sorafenib via Zebrafish Drug Screening Platform. Cancers.

[35] Wu J.Q., Fan R.Y., Zhang S.R., Li C.Y., Shen L.Z., Wei P., He Z.H., He M.F. (2020). A systematical comparison of anti-angiogenesis and anti-cancer efficacy of ramucirumab, apatinib, regorafenib and cabozantinib in zebrafish model. Life Sci..

[36] Kilarski W.W., Jura N., Gerwins P. (2003). Inactivation of Src family kinases inhibits angiogenesis in vivo: Implications for a mechanism involving organization of the actin cytoskeleton. Exp. Cell Res..

[37] Mouhayar E., Durand J.-B., Cortes J. (2013). Cardiovascular toxicity of tyrosine kinase inhibitors. Expert Opin. Drug Saf..

[38] Breccia M., Molica M., Alimena G. (2014). How tyrosine kinase inhibitors impair metabolism and endocrine system function: A systematic updated review. Leuk. Res..

[39] Lodish M.B., Stratakis C.A. (2010). Endocrine side effects of broad-acting kinase inhibitors. Endocr. Relat. Cancer.

[40] Torino F., Corsello S.M., Longo R., Barnabei A., Gasparini G. (2009). Hypothyroidism related to tyrosine kinase inhibitors: An emerging toxic effect of targeted therapy. Nat. Rev. Clin. Oncol..

[41] Lutterbeck C.A., Kern D.I., Machado Ê.L., Kümmerer K. (2015). Evaluation of the toxic effects of four anti-cancer drugs in plant bioassays and its potency for screening in the context of waste water reuse for irrigation. Chemosphere.

[42] Chen Y., Tang H., Wang L., He J., Guo Y., Liu Y., Liu X., Lin H. (2018). Fertility Enhancement but Premature Ovarian Failure in esr1-Deficient Female Zebrafish. Front. Endocrinol..

[43] Trant J.M., Gavasso S., Ackers J., Chung B.-C., Place A.R. (2001). Developmental expression of cytochrome P450 aromatase genes (CYP19a and CYP19b) in zebrafish fry (Danio rerio). J. Exp. Zool..

[44] Caballero-Gallardo K., Olivero-Verbel J., Freeman J.L. (2016). Toxicogenomics to Evaluate Endocrine Disrupting Effects of Environmental Chemicals Using the Zebrafish Model. Curr. Genom..

[45] Lin J.C., Hu S., Ho P.H., Hsu H.J., Postlethwait J.H., Chung B.C. (2015). Two zebrafish hsd3b genes are distinct in function, expression, and evolution. Endocrinology.

[46] Lepesheva G.I., Waterman M.R. (2007). Sterol 14α-demethylase cytochrome P450 (CYP51), a P450 in all biological kingdoms. Biochim. Biophys. Acta.

[47] Manchado M., Infante C., Asensio E., Planas J.V., Cañavate J.P. (2008). Thyroid hormones down-regulate thyrotropin β subunit and thyroglobulin during metamorphosis in the flatfish Senegalese sole (*Solea senegalensis* Kaup). Gen. Comp. Endocrinol..

[48] Huang G., Tian X., Fang X., Ji F. (2016). Waterborne exposure to bisphenol F causes thyroid endocrine disruption in zebrafish larvae. Chemosphere.

[49] Power D., Llewellyn L., Faustino M., Nowell M., Björnsson B.T., Einarsdottir I., Canario A.V., Sweeney G. (2001). Thyroid hormones in growth and development of fish. Comp. Biochem. Physiol. Part C Toxicol. Pharmacol..

[50] Campinho M.A., Saraiva J., Florindo C., Power D.M. (2014). Maternal Thyroid Hormones are Essential for Neural Development in Zebrafish. Mol. Endocrinol..

[51] Jin S., Antinore M.J., Lung F.D.T., Dong X., Zhao H., Fan F., Colchagie A.B., Blanck P., Roller P.P., Fornace A.J. (2000). The GADD45 inhibition of Cdc2 kinase correlates with GADD45-mediated growth suppression. J. Biol. Chem..

[52] Wang X.W., Zhan Q., Coursen J.D., Khan M.A., Kontny H.U., Yu L., Hollander M.C., O’Connor P.M., Fornace A.J., Harris C.C. (1999). GADD45 induction of a G2/M cell cycle checkpoint. Proc. Natl. Acad. Sci. USA.

[53] Zhan Q. (2005). Gadd45a, a p53- and BRCA1-regulated stress protein, in cellular response to DNA damage. Mutat. Res. Fundam. Mol. Mech. Mutagen..

[54] Drissi R., Chauvin A., McKenna A., Lévesque D., Blais-Brochu S., Jean D., Boisvert F.M. (2018). Destabilization of the MiniChromosome Maintenance (MCM) complex modulates the cellular response to DNA double strand breaks. Cell Cycle.

[55] Youle R.J., Strasser A. (2008). The BCL-2 protein family: Opposing activities that mediate cell death. Nat. Rev. Mol. Cell Biol..

[56] Hsu F.T., Sun C.C., Wu C.H., Lee Y.J., Chiang C.H., Wang W.S. (2017). Regorafenib Induces Apoptosis and Inhibits Metastatic Potential of Human Bladder Carcinoma Cells. Anticancer. Res..

[57] Tsai J.-J., Pan P.-J., Hsu F.-T. (2017). Regorafenib induces extrinsic and intrinsic apoptosis through inhibition of ERK/NF-κB activation in hepatocellular carcinoma cells. Oncol. Rep..

[58] Elmore S. (2007). Apoptosis: A Review of Programmed Cell Death. Toxicol. Pathol..

